# Magnetic Properties Improvement of Die-upset Nd-Fe-B Magnets by Dy-Cu Press Injection and Subsequent Heat Treatment

**DOI:** 10.1038/srep38335

**Published:** 2016-12-06

**Authors:** Zexuan Wang, Jinyun Ju, Jinzhi Wang, Wenzong Yin, Renjie Chen, Ming Li, Chaoxiang Jin, Xu Tang, Don Lee, Aru Yan

**Affiliations:** 1 Key Laboratory of Magnetic Materials and Devices, Ningbo Institute of Material Technology and Engineering, Chinese Academy of Sciences, Ningbo 315201, China; 2 University of Chinese Academy of Sciences, Beijing 100049, China; 3 Ningbo University of Technology, Ningbo 315211, China; 4 University of Dayton, Dayton, Ohio 45469, USA

## Abstract

Ultrafine-grained die-upset Nd-Fe-B magnets are of importance because they provide a wide researching space to redesign the textured structures. Here is presented a route to obtain a new die-upset magnet with substantially improved magnetic properties. After experiencing the optimized heat treatment, both the coercivity and remanent magnetization of the Dy-Cu press injected magnets increased substantially in comparison with those of the annealed reference magnets, which is distinct from the reported experimental results on heavy rare-earth diffusion. To study the mechanism, we analyzed the texture evolution in high-temperature annealed die-upset magnets, which had significant impact on the improvement of remanent magnetization. On basis of the results, we find that the new structures are strongly interlinked with the initial structures. With injecting Dy-Cu eutectic alloy, an optimized initial microstructure was achieved in the near-surface diffused regions, which made preparations for the subsequent texture improvement. Besides, the Dy gradient distribution of near-surface regions of the Dy-Cu press injected magnets was also investigated. By controlling the initial microstructure and subsequent diffusion process, a higher performance magnet is expected to be obtained.

Nd-Fe-B-based permanent magnets have recently regained much attention owing to the growing demand for energy efficient technologies in modern industry^1^,^2^. Maximum energy product (*BH*)_max_ directly determines the available magnetic field provided by magnet, which is closely related to both the remanent magnetization *M*_r_ and coercivity *H*_c_. In fact, field-induced alignment processing and sintering have been a mature route to achieve bulk magnets with a large (*BH*)_max_^3^, however, it is a challenge to realize large (*BH*)_max_ in die-upset Nd-Fe-B magnets prepared by melt-spun isotropic powders. For the deformation process, the growth of platelet-shaped Nd_2_Fe_14_B grains and the formation of highly textured structures have become the significant features^4^. Restricted by the inhomogeneous microstructure of starting melt-spun ribbons, the highly textured platelet-shaped grains usually coexist with the unfavorable non-aligned equiaxial grains.

For higher temperature stability, the *H*_c_ of Nd-Fe-B magnets is commonly improved by the addition of heavy rare-earth elements (HRE) into the starting alloys to obtain (Nd,HRE)_2_Fe_14_B phase with a higher anisotropy field *H*_A_^5^,^6^, which is always accompanied by the expense of the *M*_r_, where the magnetization of the HRE-substituted magnets decreases substantially due to the anti-ferromagnetic coupling of HRE and Fe moments. The limited natural abundance and the rapidly increasing cost of HRE elements have led to the need for an urgent development of less HRE-containing magnets with high *H*_c_. To decrease the usage of HRE and minimize the *M*_r_ loss, the grain boundary diffusion process (GBDP) using HRE and HRE compounds is widely studied to prepare high-coercivity sintered magnets^7^,^8^,^9^,^10^,^11^,^12^. The die-upset magnets with an ultrafine-grained structure are believed to be a potential to obtain higher *H*_c_. Actually, the value of *H*_c_ is too low to be consistent with traditional theory of the dependence of the *H*_c_ on grain size. Using three-dimensional atom probe (3DAP), a high content of Fe and Co was demonstrated in intergranular phases, as reported by Sepehri-Amin *et al*.^13^. It is believed that the strong ferromagnetism can not hinder the propagation of a reversed domain to the neighboring grains^13^,^14^,^15^,^16^. To dilute the ferromagnetism of intergranular phases and enhance the magnetic isolation of neighboring grains, the rare-earth eutectic alloys were commonly infiltrated into die-upset magnets, such as Nd-Cu^16^,^17^,^18^,^19^,^20^, Pr-Cu^20^,^21^, (Nd, Dy)-Cu^22^. Unfortunately, the infiltrated rare-earth eutectic alloys decrease the proportion of the main phase and deteriorate the highly textured structures. Therefore, the remarkable reduction of *M*_r_ accompanying of enhancing *H*_c_ is a widespread phenomenon for Nd-Fe-B-based bulk materials^16^,^18^,^19^,^21^,^22^,^23^. There seems to be an impassable gulf separating the *H*_c_ enhancement from the *M*_r_ improvement in one system. How to enhance *M*_r_ and *H*_c_ in Nd-Fe-B-based bulk materials simultaneously to prepare high-performance magnets has become a long-term unsolved critical issue.

In this work, we investigated the mechanism of texture improvement in annealed die-upset magnets and revealed direct correlation between the new structure and the initial structure. By injecting Dy-Cu eutectic alloy, the magnets with an optimized initial microstructure were obtained, which made preparations for further texture improvement during the heat treatment. In addition, the Dy diffusion process was accelerated because of the high-temperature annealing treatment, resulting in a higher *H*_A_ in the (Nd, Dy)_2_Fe_14_B shell structure.

## Results

### Magnetic properties

**Figure 1:**
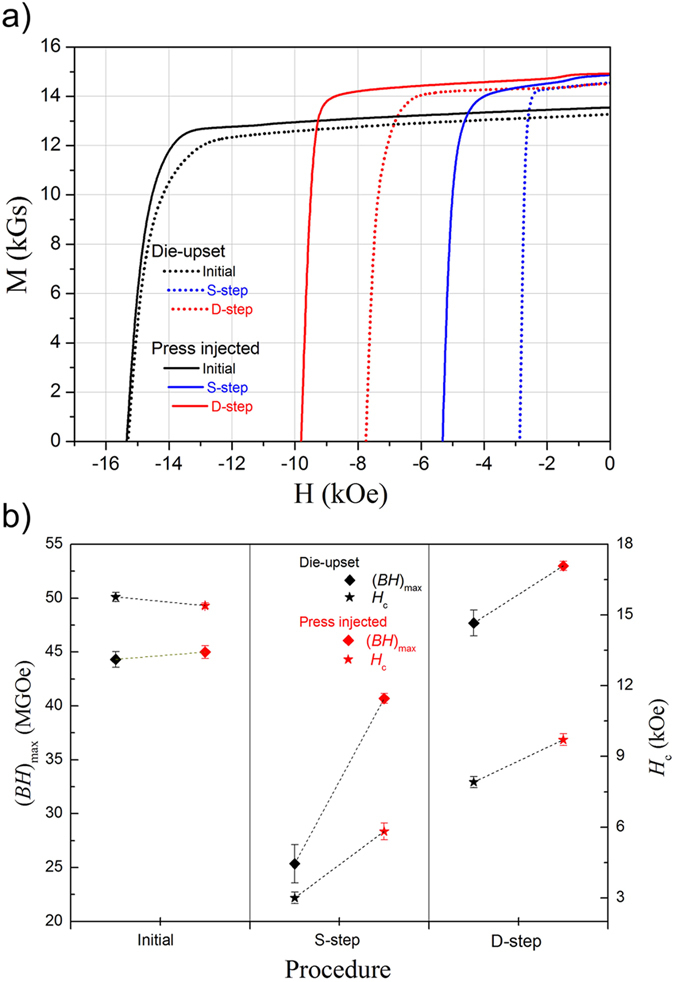
(**a**) Demagnetization curves of the die-upset and Dy-Cu press injected magnets as well as their individual annealed magnets, (**b**) Magnetic properties of the die-upset and Dy-Cu press injected magnets at different stages of the procedure: initial state, S-step and D-step annealed states.

Figure 1a shows the demagnetization curves of the die-upset (reference) and Dy-Cu press injected magnets as well as their individual annealed magnets. Hereafter, the annealing at *T*_high_ = 900 °C for 120 min is denoted as single-step heat-treatment (S-step), while annealing at *T*_high_ = 900 °C and *T*_low_ = 700 °C for 120 min successively as dual-step heat-treatment (D-step). Once prepared by press injection process, the magnetic properties of the injected magnets are analyzed, almost equivalent to those of die-upset magnets. Then the value of *M*_r_ is enhanced to 14.5 kGs for die-upset magnets, 14.9 kGs for press injected magnets after the single-step annealing treatment. The injected non-ferromagnetic Dy-Cu liquid markedly reduces the *H*_c_ degradation owing to the enhanced anisotropy field *H*_A_ of (Nd, Dy)_2_Fe_14_B phase and non-ferromagnetism of the intergranular phases^2^,^22^,^24^. Still, the *H*_c_ deteriorates too seriously to obtain a favorable (*BH*)_max_. After a low-temperature annealing treatment, the *H*_c_ are recovered to 7.7 kOe and 9.7 kOe for the die-upset and press injected magnets, respectively. Due to the microstructure optimization exerted under a lower temperature, their *H*_c_ is improved without sacrificing the enhanced *M*_r_. As a consequence, the values of (*BH*)_max_ are recovered to 48 MGOe for the die-upset magnets, and 53 MGOe for the press injected magnets. Interestingly, the *M*_r_, *H*_c_ and (*BH*)_max_ of the press injected magnets annealed by single-step or dual-step annealing treatment are markedly higher than those of the reference magnets upon the same heat treatment (Fig. 1b).

### Remanent magnetization enhancement and its key figure of merit

**Figure 2:**
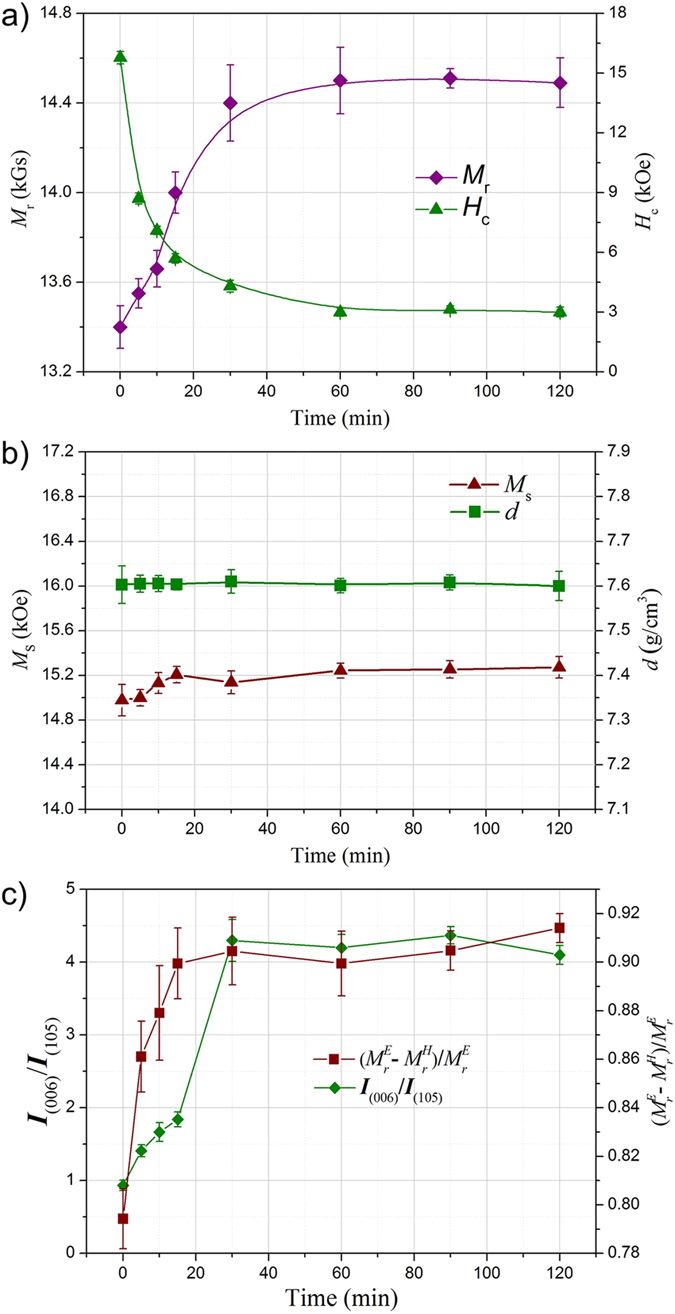
(**a**) Remanent magnetization *M*_r_, coercivity field *H*_c_, (**b**) saturation magnetizations *M*_s_, densities *d* and (**c**) orientation degrees of the die-upset Nd-Fe-B magnet as a function of the high-temperature annealing time.

Obviously, the high-temperature heat treatment plays a significant role in the enhancement of *M*_r_. To avoid the uncontrollable influence of injected Dy-Cu liquid on the microstructure, the investigation of *M*_r_ enhancement is established on reference die-upset magnets. The *M*_r_ rapidly increases from 13.4 to 14.4 kGs, and then reaches a maximum after annealed for 30 min (Fig. 2a). The *H*_c_ exhibits the opposite behavior, decreased from 15.6 to 3.0 kOe. This suggests that significant microstructure evolution might take place in the initial phase of the high-temperature heat treatment, leading to the *M*_r_ improvement. To explore the nature of the improved *M*_r_, some parameters should be taken into consideration according to the law for *M*_r_^25^:


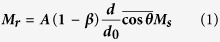


where *A* is the volume fraction of the positive domain parallel to the pre-magnetization, (1 − *β*) the volume fraction of hard magnetic phase, *d*_0_ the theoretical density, *d* the actual density, 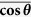 the orientation degree and *M*_s_ the saturation magnetization of magnets.

As the *A* directly reflects the reversed magnetized ability of pre-magnetized magnets, the declined *H*_c_ of the annealed magnets suggests that the propagation of a reversed domain becomes easier. The increased reversed domains are not in conformity with the tendency of the improved *M*_r_. In addition, no non-ferromagnetic additives are added to or removed from the reference system. Therefore, the parameters *A* and *β* are not the major factors for the equation (1). For the die-upset magnets annealed before and after, their densities *d* are nearly invariable, ~7.6 g/cm^3^, as shown in Fig. 2b. The value of saturation magnetizations *M*_s_ (the magnetization measured using PPMS at a maximum applied field of 90 kOe) is subject to a slightly monotonous increase from 15.0 to 15.2 kOe within 30 min. However, the *M*_s_ just increases by 1.4%, which is also insufficient to support the increased *M*_r_, about 7.5%. Based on above analysis, the orientation degree of annealed magnets seems to be the most likely reason for the *M*_r_ enhancement. The exact values of their orientation degrees are numerical calculated by the intensity ratio of (006)/(105) peaks in the XRD measurements and *M*_r_ ratio 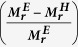, where 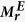 and 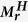 are the remanent magnetization parallel and perpendicular to the easy-axis, respectively (Fig. 2c). Both calculated values increase rapidly within 30 min, and ultimately reach a maximum. This tendency is consistent with the result on *M*_r_ evolution (Fig. 2a). From the above discussion, a general rule can be drawn that the *M*_r_ enhancement taking place within a narrow time range mainly benefits from the optimized orientation degree in the annealed die-upset magnets.

### Microstructure evolution and texture investigation

**Figure 3:**
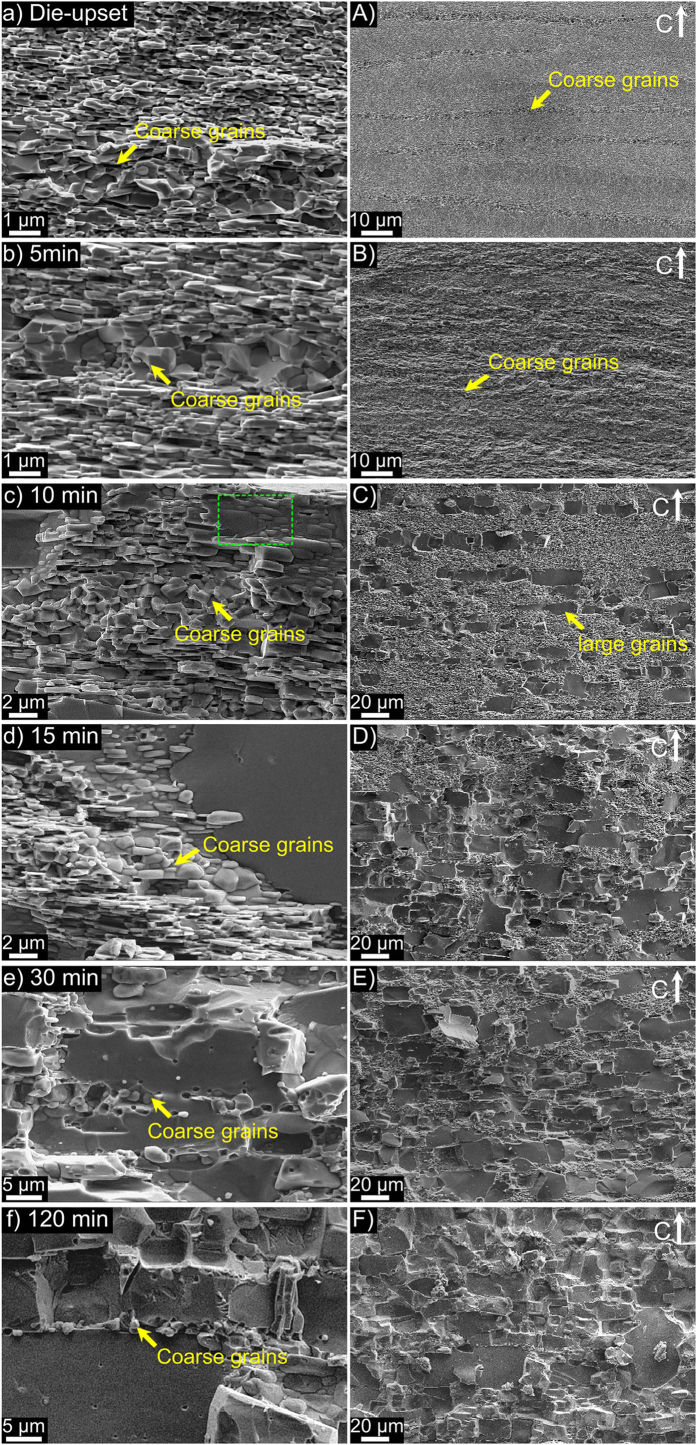
SEM images of morphologies of the die-upset magnet (**a**) and its magnets annealed at 900 °C for 5 (**b**), 10 (**c**), 15 (**d**), 30 (**e**) and 120 min (**f**); (A–F) show broader areas corresponding to the same magnets for (**a**–**f**), respectively. (*c*-axes parallel to the arrow directions).

For the annealed die-upset magnets with a remarkable *M*_r_ enhancement, we measured their microstructures to determine crystal growth of the Nd_2_Fe_14_B phase. In untreated initial magnets, two layers with boundaries are visible: the former consists of well-aligned platelet-shaped Nd_2_Fe_14_B grains and the latter is composed of non-aligned equiaxial Nd_2_Fe_14_B grains, as shown in Fig. 3a. Both described layers are distributed alternately on a larger scale, with 10–25 μm apart^26^ (Fig. 3A). Furthermore, the platelet-shaped Nd_2_Fe_14_B grains with an average size of 500 nm in length, 80 nm in thickness is observed, smaller than that of the coarse grains. After annealed for 5 min (Fig. 3b), the morphology remains an evident platelet-shaped structure, where two kinds of grains had grown up simultaneously. However, the interfaces between the two layers are easier to be observed. The growth of non-aligned coarse grains accelerates the extension of coarse grain layers, leading to a slight increase of their thickness. The thickened coarse grain layers are unfavorable for the *M*_r_ enhancement, likely to threaten the highly textured structure. In fact, however, the value of the *M*_r_ increased from 13.4 to 13.6 kOe. Thus, the dominant factor of *M*_r_ enhancement has close relevance to the microstructure evolution taking place in the well-aligned layers rather than the non-aligned layers. With extending the annealing time, this phenomenon of the observed thickened non-aligned layers is suppressed. The growth rate in the platelet-shaped grain layers is superior to that of the coarse grain layers. Partial well-aligned platelet-shaped grains tend to form micron-scale grains side by side, while the sizes of the coarse grains are not significant changed any more, as shown in Fig. 3c and C. It’s worth noting that a few regions with illegible grain boundaries are also observed in platelet-shaped layers, marked by the dashed box in Fig. 3c, which are supposed to be the precursors for the large grains. With further increasing the time of annealing treatment, the coarse grain layers are gradually devoured by neighboring micron-scaled grains to form larger Nd_2_Fe_14_B grains (Fig. 3d–f). Based on the inhomogeneous grain growth, the initial textured structure obviously has an essential impact on the following microstructures, leading to a preferential growth in the platelet-shaped layers and suppresses the abnormal growth of non-aligned coarse grains^27^.

**Figure 4:**
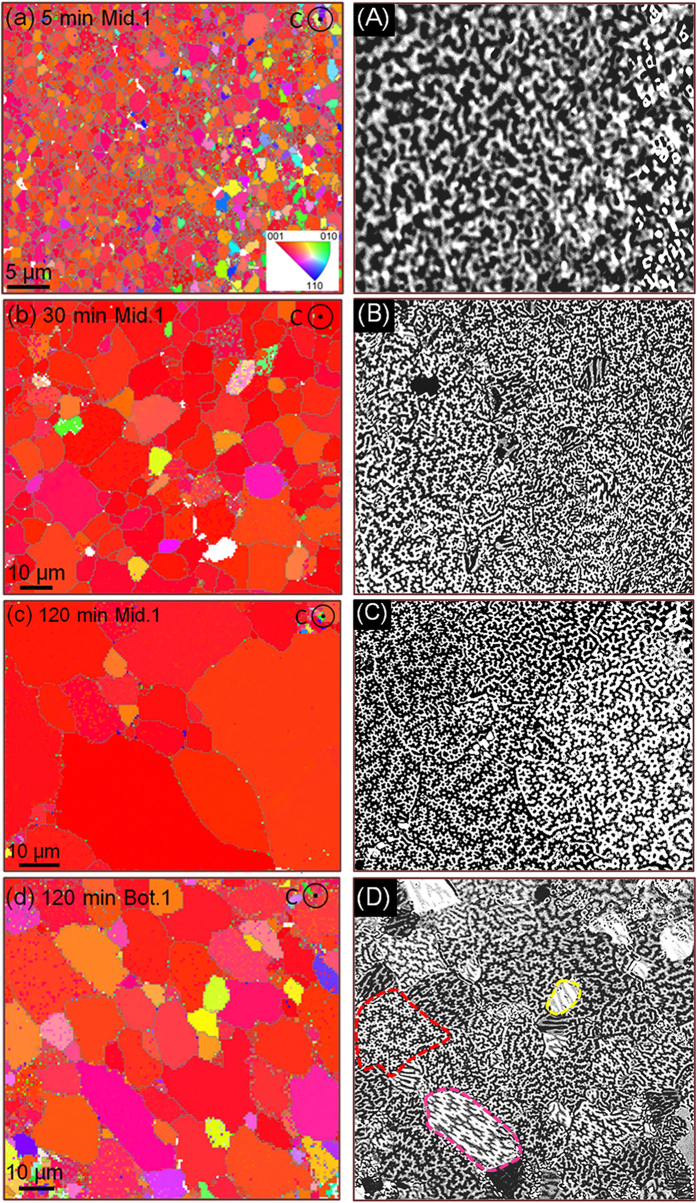
EBSD maps of the middle (Mid.1) and near-surface (Bot.1) cross-sections parallel to the basal planes of the die-upset magnets annealed at 900 °C for 5 (**a**), 30 (**b**) and 120 min (**c**,**d**). Magneto-optical Kerr domains (A–D) corresponding to the very same regions in (**a**–**d**), respectively. (*c*-axes perpendicular to the basal planes, the unidentified areas are marked with white color).

From above discussion, the new platelet-shaped structure after annealing is strongly interlinked with the initial structure. Due to the complicated rheological behavior taking place in the deformation, there is reason to believe that textures in annealed die-upset magnets are also remarkably inhomogeneous. This motivates us to investigate the texture evolutions in annealed magnets systemically. By means of the EBSD technology, one can find that the texture of the middle area (Mid.1) on the basal plane (Fig. 4a–c) is optimized with increasing annealing time, where the number of misoriented grains is decreasing. In addition, the texture of the middle area is obviously superior to that of the bottom area (Fig. 4c and d). The relatively disordered alignment in latter has close relevance to the initial structure, which is commonly considered as a low textured regions. However, its grain size is smaller in comparison with the former. The slowing grain growth is supposed to be inhibited by the relatively disordered initial texture, where the microstructures lack the predominant orientation.

As a typical character of the microstructure of magnetic materials, magnetic domain links the intrinsic physical properties of a material with its macroscopic properties. To establish the inner relation of texture evolutions with domain structures, we studied the domain structures in the thermally demagnetized state at the very same regions of EBSD maps, as shown in Fig. 4A–D. For the magnet annealed with 5 min, a maze domain structure without branching is clearly exhibited on the well-aligned area, which is commonly seen in the highly textured die-upset magnets^28^,^29^,^30^. The typical feature size of an average domain width is about 1 μm, similar to the size of the growing Nd_2_Fe_14_B grains (Fig. 4a). When extending annealing time (Fig. 4B), a so-called star-like domain is captured on micron-scaled grains with a size of about 7–15 μm, which is a typical domain pattern exhibited on the basal plane of large Nd_2_Fe_14_B grains^31^. This branched domain patterns are mainly attributed to the effective thickness *D* of the surface grains, which will not be further discussed here. There are a few stripe-like and transition domain shown on the residual misoriented grains. With further grain growth, the branching degree of the domain patterns increases (Fig. 4C). Hence, the branching structure of magnetic domains can be considered as a significant symbol for texture optimization in this work. In addition to grain size, the basic domain structure is also sensitive to the orientation angle of an Nd_2_Fe_14_B grain because of its high uniaxial anisotropy. For the Bot.1 area (Fig. 4D), various branching domain structures are observed on the random oriented micron-scale grains, such as the star-like (red dotted area), stripe-like (yellow dotted area) and transition domains (pink dotted area). When [00l] axes of Nd_2_Fe_14_B surface grains deviating from the observation direction gradually, the star-like, transition and stripe-like domains will be obtained in turn. By studying the domain structure, a better texture is easier to be achieved in the initial highly textured regions rather than the disordered regions. Hence, we can conclude that the new structures are strongly interlinked with the initial structures. This conclusion is supported by the result reported by A. Hubert and R. Schäfer, where a systematic series of domain patterns on Nd_2_Fe_14_B crystals as a function of orientation angles are established^31^.

**Figure 5:**
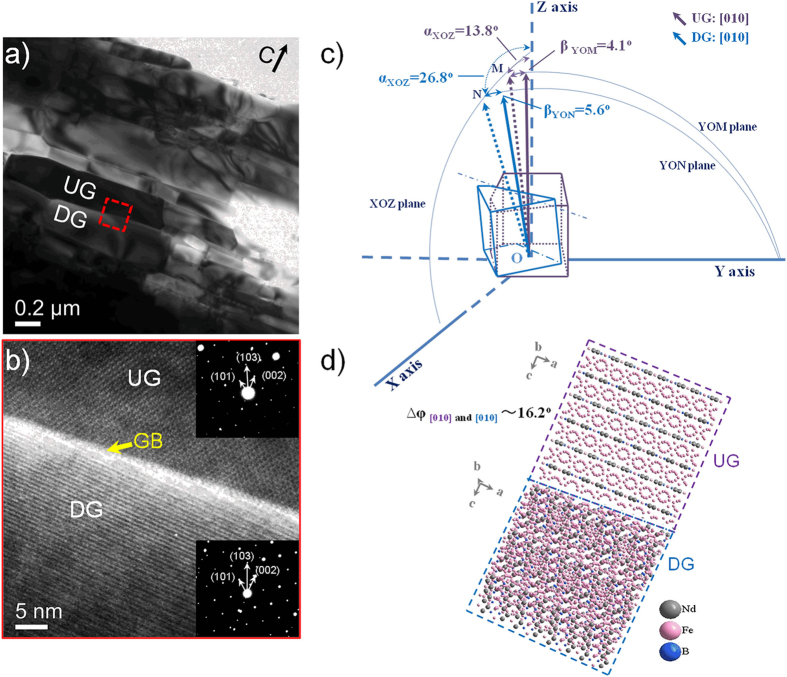
(**a**) BF TEM image of the die-upset magnet after annealed at 900 °C for 5 min. High-resolution TEM (**b**), geometric model (**c**) and crystal structure (**d**) of the selected grain boundary corresponding to the red dashed region in (**a**). (The two inserts in (**b**) are diffraction patterns along [0l0] crystal zone axes of UG and DG, respectively).

For the annealed magnets, the driving force for the preferential growth of the platelet-shaped grains has become a critical issue. In our previous work^32^, the mechanism of grain growth and texture development in nanocomposite magnets has been reported. The grains with their *c*-axes closer to the applied stress are supposed to own a little more energetically favorable. Therefore, it could be concluded that the gap of the strain energy of two neighboring Nd_2_Fe_14_B grains might be a dominate factor for the stress-induced aligned magnets. Different from the stress-induced preferential growth, the grain growth taking place in this work is only constrained by the initial platelet-shaped structure of the die-upset magnet. To reveal the driving force in the high-temperature process, the intermediate state of a magnet annealed at 900 °C for 5 min has been investigated (Fig. 5). It can be seen that a number of platelet-shaped grains with a grain size of ~1000 nm in length, ~250 nm in thickness is observed, at least twice larger than that of the initial grains. Besides, in many grain boundaries, a number of lattice overlapping is observed due to the absence of the Nd-rich intergranular phase, as shown in Fig. 5b. To clarify the lattice mismatch at the grain boundary, we attempt to identify the orientation relationship of the neighboring platelet-shaped Nd_2_Fe_14_B grains (denoted as Up Grain and Down Grain, hereafter UG and DG for short) in three-dimensional space. The spatial orientation of each grain is identified by rotating TEM specimen holder to achieve the modest kikuchi bands and the diffraction patterns. After rotating the ultrathin specimen, the electron beam incident direction is parallel to [0l0] crystal zone axis of UG and DG, respectively. Based on the starting position of the inserted specimen (α = 0°, β = 0°), the rotation angles along two independent directions could be evaluated by the values of α and β. When observed along their individual [0l0] crystal zone axis, α = 13.8°, β = 4.1° and α = 26.8°, β = 5.6° are obtained for UG and DG, respectively. To reconstruct the spatial geometry models in XYZ-O coordinates (Fig. 5c), we suppose α is the angle that the [0l0] crystal zone axis of UG (the violet dotted arrows) and DG (the blue dotted arrows) deviates from Z-axis in XOZ plane, and β is the angle that their rotated [0l0] crystal zone axis deviates from the XOZ plane on the basis of the dotted arrows. Finally, their [0l0] crystal zone axes are identified in the three-dimensional geometric model (the violet and blue solid arrows). In addition, the actual spatial angle ∆φ between their [0l0] crystal zone axes is also calculated by the values of α and β, approximately 16.2°. To simplify the crystal structure model, the observation direction is parallel to the [0l0] crystal zone axis of the UG (the region marked with violet dotted box). Therefore, the corresponding crystal structure model for the neighboring Nd_2_Fe_14_B grains is described, as shown in Fig. 5d. Due to the absence of intergranular phases, neighboring grains directly contact with each other. The lattice mismatches existing at the interfaces of grain boundaries result in a large amount of lattice distortion. When annealed at a relatively low temperature, the Nd_2_Fe_14_B grains grow up rapidly. The lattice mismatches are gradually eliminated by the growth of Nd_2_Fe_14_B grains. The lattice distortion is also released owing to the reduction of lattice mismatches. Obviously, the underlying unsteady structure makes preparations for structural optimization. Thus, the minimization of the lattice distortion stored in the grain boundaries might be a key to driving the microstructure evolution in the high-temperature annealing system.

### Influence of Dy-Cu press injection and subsequent heat treatment on the microstructures

**Figure 6:**
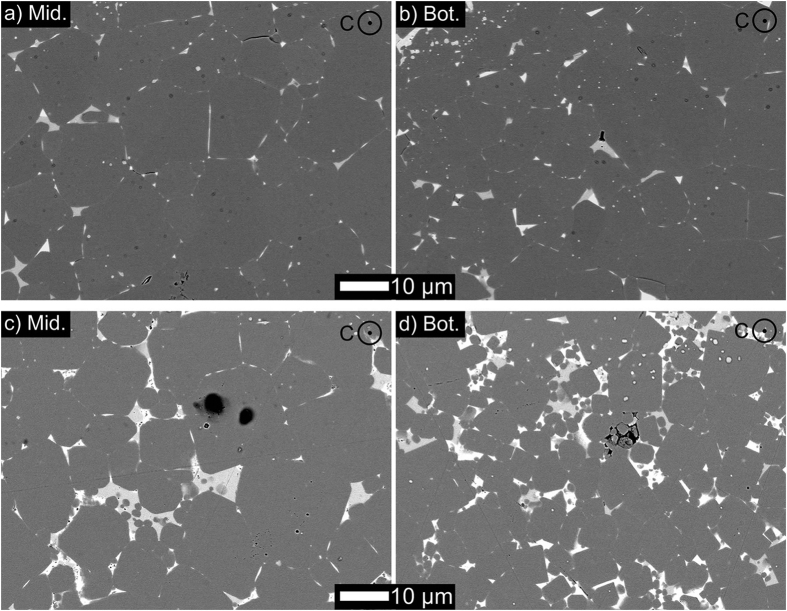
BSE SEM images of the middle (Mid.) and near-surface (Bot.) cross-sections parallel to the basal planes of the die-upset (**a**,**b**) and Dy-Cu press injected (**c**,**d**) magnets after the D-step annealing treatment.

Due to the Dy-Cu eutectic liquid injected by applied pressure, the precursors with an optimized texture are obtained in the injected magnets. The Dy-Cu eutectic alloy should play a critical role in the microstructure evolution. The microstructures of different areas in the annealed die-upset and Dy-Cu press injected magnets exhibit remarkable difference. For the Mid. area in annealed die-upset magnets (Fig. 6a), some Nd_2_Fe_14_B grains were separated by a thin Nd-rich intergraular phase with a bright contrast. Due to the grain growth, the rare-earth-rich intergranular phases were aggregated to triple junction areas. Hence, a few large triple-junction phases were observed in the large grain-sized area. Compared with the Mid. area, many grains with a smaller size were captured on the Bot. area (Fig. 6b). In addition, the distribution of triple-junction phases is also improved, where smaller triple-junction phases are frequently observed. With injecting Dy-Cu liquid, a few aggregated ultra-large rare-earth-rich phases are exhibited on the Mid. area, where smaller main grains are isolated by these aggregations. This phenomenon is markedly enhanced on the near-surface Dy-Cu injected area (Fig. 6d). There is a pronounced decrease of Nd_2_Fe_14_B grain size with injecting Dy-Cu eutectic liquid. The reduction of the grain size and the thickened intergranular phase is favorable for the *H*_c_ improvement. For the further enhanced *M*_r_, a higher textured structure might be obtained on the near-surface Dy-Cu injected area, and this optimized structure is inherited by subsequent high-temperature annealed magnets. It could be concluded that the Dy-Cu press injection introduced in the deformation procedure accelerates the texture formation of the near-surface regions.

**Figure 7:**
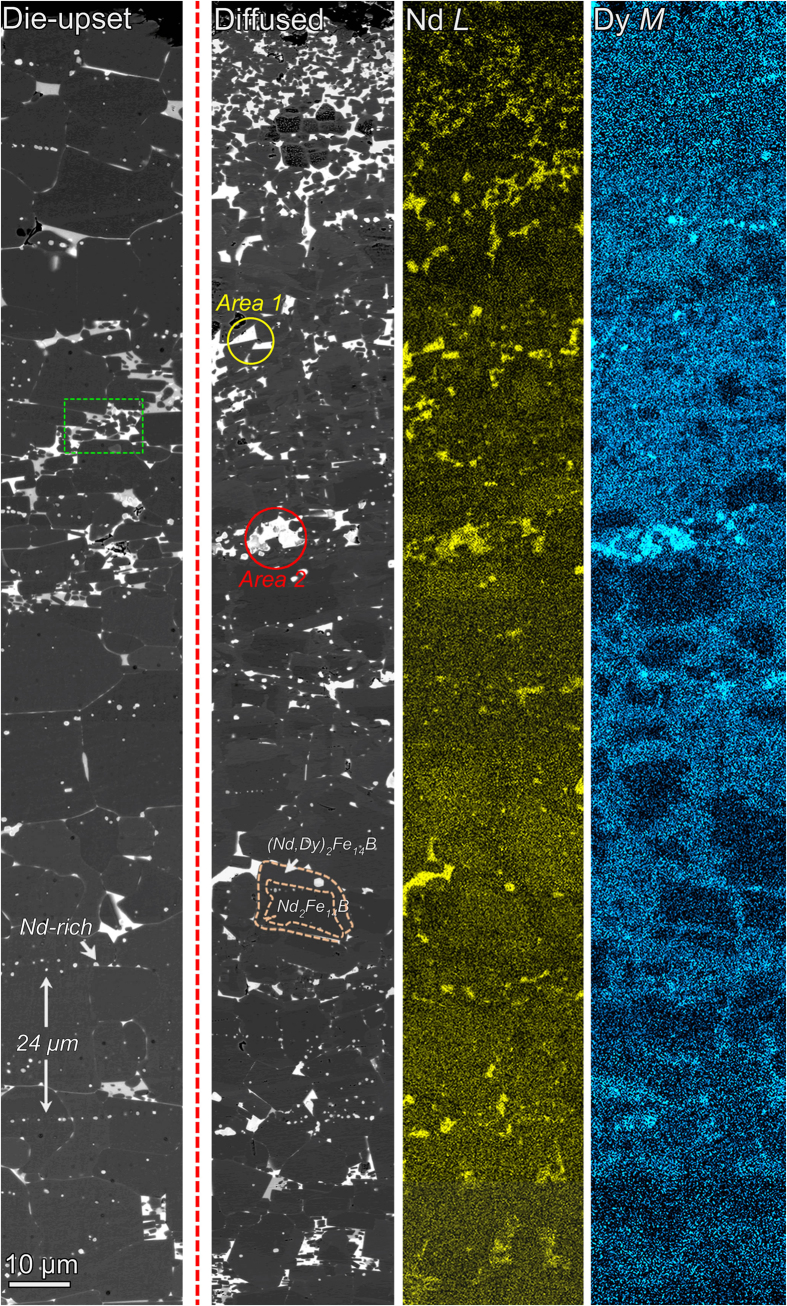
BSE SEM images of near-surface vertical-sections of the die-upset and Dy-Cu press injected magnets after the D-step annealing treatment, and EDX maps of Nd and Dy elements for the annealed injected magnet; two rare-earth-rich areas with different Nd/Dy ratios are marked by circles. (*c*-axes parallel to the observation sections).

To investigate the Dy diffusion gradient established by the press injection process and subsequent heat-treatment, BSE images and EDX maps of Dy and Nd elements were performed on the near-surface microstructures of die-upset and diffusion-processed magnets processed by dual-step annealing treatment, as shown in Fig. 7. A remarkable large grain-sized structure with residual nano-scale and sub-micron grains is observed in both annealed magnets. Most of large grains with a rectangular shape suggest that the new structure, derived from the platelet-shaped nano-structure, is significantly different from the sintered magnets^7^,^8^,^9^. In annealed die-upset magnets, a series of nano-scale Nd-rich aggregations are found inside large Nd_2_Fe_14_B grains, and the distance of neighboring aggregations is about 24 μm, similar to the thickness of deformed melt-spun ribbons. Based on the investigation for grain growth, the residual aggregations are supposed to the intergranular phases enriched at the interfaces of ribbons, which are not removed in time. In the aggregated Nd-rich triple-junction phases, we can also find the isolated grains have a smaller grain size (marked with green dotted box). The Nd-rich phases are helpful for inhibiting grain growth.

When injected Dy-Cu liquid by applied pressure, the microstructure of annealed injected magnets exhibit distinct differences. The grains in the vicinity of the diffused surface show a remarkable (Nd, Dy)_2_Fe_14_B/Nd_2_Fe_14_B shell-core structure, leading to an enhanced *H*_A_ in the Dy-enriched shell. The Nd_2_Fe_14_B grains with Dy substitution showed an enhanced contrast owing to the higher atom number of Dy atom. The thickness of the (Nd, Dy)_2_Fe_14_B shell decreases rapidly with increasing diffusion depth and is visible up to a depth of more than 600 μm. After analyzing the rare-earth-rich grain boundary phases, we also find that two large areas with different Nd/Dy ratios are distinguished (denote as Area 1 and Area 2). Obviously, there is a higher Dy content in Area 2 in comparison with Area 1. Besides the formation of (Nd, Dy)_2_Fe_14_B/Nd_2_Fe_14_B shell-core structure and the ratios of Nd/Dy atoms in grain boundary phases, the injected Dy-Cu liquid has significant impact on grain growth of near-surface injected area. More grains are isolated by thicker Nd(Dy)-rich intergranular phases and their growth is largely suppressed during the subsequent heat treatment. By reducing the grain size and thickening the Nd(Dy)-rich intergranular phases, the microstructure has potential to provide more pinning sites to inhibit magnetic domain wall motion. Meanwhile, the largest value of stray field usually exists at the interfaces of the surface grains also decrease due to the reduction of surface grain size^33^. Therefore, this microstructure with gradient grain sizes might be another contribution to the improved *H*_c_ for the annealed diffusion-processed magnets^34^. By the press injection technology, the shape anisotropy of the surface grains is enhanced, implying that the deformability of near-surface grains is optimized. This also makes better preparations for the preferential growth of Nd_2_Fe_14_B grains. Thus, the values of *H*_c_, *M*_r_ and (*BH*)_max_ of annealed diffusion-processed magnets are improved simultaneously.

## Discussion

Due to stress-induced alignment process, there are a large number of crystallographic differences in neighboring Nd_2_Fe_14_B grains. On the other hand, lower rare-earth content results in the direct contact of neighboring grains, where a number of lattice distortion exists at the interfaces. The imperfection suggests the underlying structural instability in die-upset magnets, which makes preparation for microstructure evolution. By optimized heat treatment, the lattice mismatches are gradually eliminated by the growth of Nd_2_Fe_14_B grains. The lattice distortion stored at the grain boundaries is also released. After studying texture evolution, we find that the ultimate textures have close relevance to the initial textures. The regions with a highly textured structure are more favorable to the preferential growth of Nd_2_Fe_14_B grains and able to bring about an improvement of magnetic properties. The non-uniform growth first takes place in the platelet-shaped grain layers. Then these large grains start devouring the coarse grains to obtain an optimized texture. Due to the grain growth, the large Nd-rich triple-junction phases are frequently observed in the annealed magnets.

In die-upset magnets, the near-surface region has a low textured structure, where many non-aligned grains are observed. With injecting Dy-Cu eutectic liquid, the deformability of the near-surface grains is optimized. The optimized initial microstructure is remarkably favorable to the following texture improvement. Besides, theses grains are isolated by injected rare-earth-rich phases. Their growth is largely suppressed by the enriched rare-earth aggregations during the heat treatment. In the high-temperature process, the diffusion of Dy element is also accelerated to form (Nd, Dy)_2_Fe_14_B phases. Finally, both the refined near-surface grains and (Nd, Dy)_2_Fe_14_B/Nd_2_Fe_14_B shell-core structure improved the *H*_c_. It can’t be ignored that a few micron-scale grains with their *c*-axes deviating from the easy-axis are also found in the annealed magnets. This implies that we can reutilize these grains by redesigning the initial microstructure of die-upset magnets to achieve a larger (*BH*)_max_.

## Conclusions

In this work, we reported a new pathway to redesign die-upset Nd-Fe-B magnets with a large (*BH*)_max_ of 53 MGOe. On basis of the research, two significant laws have been summarized. Firstly, the texture of the die-upset magnets after experiencing dual-step heat treatment could be substantially improved, which led to a higher *M*_r_. Interestingly, the new microstructure had close relevance to its initial microstructure. A better annealed texture was easier to be obtained in the well-aligned region rather than the disordered region. Thus, the starting magnets with an optimized texture are expected to achieve higher performance magnets. Secondly, the near-surface microstructure of die-upset magnets was remarkably improved due to the injected Dy-Cu eutectic alloy. It also makes preparations for the further texture optimization during the subsequent heat treatment. The (Dy,Nd)-rich liquid largely suppressed the near-surface grain growth and accelerated the formation of (Nd, Dy)_2_Fe_14_B/Nd_2_Fe_14_B shell-core structures. Finally, the improved *M*_r_ and *H*_c_ were obtained simultaneously in the annealed diffusion-processed magnets.

Furthermore, this unique technique breaks through the existing definition for bulk Nd-Fe-B-based magnets by grain size. It may be not only restricted in the die-upset magnets and can be generalized to other well-aligned ultrafine-grained Nd-Fe-B-based systems. We also hope this work can evoke to redesign microstructure in highly textured ultrafine-grained magnets, opening a brand new door to understand the characters of Nd-Fe-B-based bulk materials.

## Methods

### Magnet preparation

Commercial melt-spun isotropic MQU-F powders with a composition Nd_30.3_Pr_0.1_Fe_64.12_Co_4.1_Ga_0.46_B_0.92_ (wt.%, measured by ICP) were purchased from Magnequench International Inc. The hot-pressed magnets with a size of 16.3 mm in diameter, 22 mm in height were prepared under the protection of high vacuum, lower than 5 × 10^−2^ Pa. Approximately 20 g of Dy_70_Cu_30_ (at. %, *T*_melting point_ = 790 °C) alloys were prepared by arc melting using high purity metal Dy and Cu. The arc melted buttons were then sliced into thin slices with a size of 16.3 mm in diameter, 0.8 mm in thickness to prepare for the following press injection process. Two thin Dy_70_Cu_30_ slices were coated on the surface of the hot-pressed magnet up and down, and then hot deformed until their height reduced by 70% under the protection of highly purified argon atmospheres. The conventional die-upset magnets without coating thin Dy_70_Cu_30_ slices were prepared by the same hot-deformed procedure. Next, all the annealing treatments were exerted under the protection of highly purified argon atmospheres, and then cooled to room temperature.

### Measurements and characterizations

The demagnetization curves were measured using a closed circuit B-H apparatus (NIM 500 C) after pre-magnetized in a pulsed field of 80 kOe. The hysteresis loops along easy and hard axes were measured using a physical property measurement system (PPMS) vibrating sample magnetometer (VSM) with a maximum applied field of 90 kOe. The peak ratio analysis was carried out by X-ray diffraction (XRD) with Cu *K*_a_ radiation. Backscattered electron (BSE) SEM observations were made on the bulk magnets using a Carl Zeiss Cross Beam equipped with an energy dispersive X-ray spectroscopy (EDX) detector. Electron backscatter diffraction (EBSD) images were picked up using Oxford plug-in to investigate texture evolution. The domain patterns were studied using magneto-optical Kerr microscope (Evico magnetics GmbH). Transmission electron microscopy (TEM) observations were performed using a Tecnai-F20 system with an acceleration voltage of 200 kV. The magnets with a size of 10 mm in diameter, 6.4 mm in height were dipped into acetone and clean with ultrasonic washer for 5 minutes, and then their densities were identified by Archimedes drainage method. The experimental data were reproducible, with no less than three cases. And their standard deviations were calculated by the formula: 
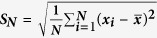
.

## Additional Information

**How to cite this article**: Wang, Z. *et al*. Magnetic Properties Improvement of Die-upset Nd-Fe-B Magnets by Dy-Cu Press Injection and Subsequent Heat Treatment. *Sci. Rep.* **6**, 38335; doi: 10.1038/srep38335 (2016).

**Publisher's note:** Springer Nature remains neutral with regard to jurisdictional claims in published maps and institutional affiliations.
